# SOD1 co-pathologies in the brain: insights from neuropathological evidence and implications

**DOI:** 10.1007/s00401-026-03090-7

**Published:** 2026-10-06

**Authors:** Evellyn Mayla de Azevedo, Victoria Simões Bernardo, Marcelo M. S. Lima, Elis Cristina Araujo Eleutherio, Tiago Fleming Outeiro

**Affiliations:** 1 Department of Experimental Neurodegeneration, Center for Biostructural Imaging of Neurodegeneration, University Medical Center Göttingen, Göttingen, Germany; 2 Neurophysiology Laboratory, Department of Physiology, Biological Sciences Center, Universidade Federal Do Paraná (UFPR), Curitiba, PR Brazil; 3 Institute of Chemistry, Universidade Federal Do Rio de Janeiro, Rio de Janeiro, RJ Brazil; 4 Faculty of Medical Sciences, Translational and Clinical Research Institute, Newcastle University, Newcastle Upon Tyne, UK; 5 Deutsches Zentrum Für Neurodegenerative Erkrankungen (DZNE), Göttingen, Germany; 6 Faculdade de Medicina E Ciências Biomédicas, Algarve Biomedical Center Research Institute, Universidade Do Algarve, Faro, Portugal

**Keywords:** Co-pathologies, ALS, FTD, Protein aggregation, Neuropathology

## Abstract

Superoxide dismutase 1 (SOD1), originally regarded as an antioxidant enzyme, is now recognized as a multifunctional protein whose conformational instability and aggregation contribute to neurodegeneration. The emerging evidence suggests that SOD1 pathology extends beyond amyotrophic lateral sclerosis, supporting a role in multiple neurodegenerative diseases. This review summarizes current neuropathological evidence of SOD1 co-pathologies in the human brain, with particular emphasis on its associations with TAR DNA-binding protein 43 (TDP-43), tau, alpha-synuclein, amyloid-β, and other aggregation-prone proteins. We examine the distribution of pathological SOD1 species across neurodegenerative diseases, their coexistence in affected brain regions, and potential mechanisms underlying these interactions, including oxidative stress, impaired proteostasis, aberrant phase separation, and cross-seeding. We also discuss emerging observations of SOD1 pathology in neurological conditions beyond classical neurodegenerative diseases, raising the possibility that SOD1 misfolding represents a broader cellular response to neuronal injury. Collectively, the available evidence supports a shift from viewing SOD1 as a disease-specific protein to considering it a potential participant in interconnected proteopathic networks. Although the functional significance of SOD1 co-pathologies remains incompletely understood, elucidating the molecular basis of aggregation may provide new insights into disease onset and progression, improving our understanding of neurodegenerative mechanisms, and facilitating the development of biomarkers and the identification of therapeutic targets across diverse proteinopathies.

## Introduction

Neurodegeneration refers to the progressive and selective loss of neuronal structure and function, ultimately leading to irreversible neuronal death and clinical decline. Major neurodegenerative diseases (NDs), including Alzheimer’s disease (AD), Parkinson’s disease (PD), Huntington’s disease (HD), amyotrophic lateral sclerosis (ALS), and frontotemporal dementia (FTD), differ in their predominant anatomical vulnerability and clinical presentation, yet converge at multiple mechanistic levels [25]. Accumulating evidence from genetic, transcriptomic, and proteomic studies indicates that these disorders share dysregulated pathways involving: (i) protein homeostasis, (ii) RNA metabolism, (iii) mitochondrial dysfunction, (iv) oxidative stress, (v) impaired axonal transport, (vi) neuroinflammation, and (vii) defective proteostasis networks [25, 60, 73]. Among these shared mechanisms, disruption of protein homeostasis has emerged as a central pathogenic event. The accumulation of misfolded and aggregation-prone proteins not only reflects impaired proteostasis but also contributes to the progressive disruption of interconnected cellular pathways, ultimately driving neuronal dysfunction and degeneration [17, 18].

Originally, neurodegenerative disorders were classified according to the predominant protein aggregates found upon neuropathological examination. The defining proteins of those aggregates, amyloid-β and tau in AD [28, 55], alpha-synuclein (aSyn) in PD [63], huntingtin in HD [21], and superoxide dismutase 1 (SOD1) or TAR DNA-binding protein 43 (TDP-43) in ALS and FTD [5, 26], are now known not to be specific for each disorder. In fact, neuropathological studies have demonstrated that different pathogenic proteins coexist within the same brain regions, cell populations or even individual neurons, suggesting extensive molecular crosstalk among distinct proteostasis components [3, 10, 44, 47, 57].

Among the various proteins implicated in neurodegeneration, SOD1 occupies a unique position because its physiological functions and pathological alterations extend across multiple neurodegenerative diseases. It was initially viewed as a ubiquitously expressed antioxidant enzyme that catalyzes the conversion of superoxide anions into molecular oxygen and hydrogen peroxide, constituting one of the first lines of cellular defense against reactive oxygen species (ROS) [40]. Therefore, SOD1 plays a fundamental role in maintaining cellular redox homeostasis, particularly in the nervous system, where neurons are highly vulnerable to oxidative damage [14, 22, 70]. However, in addition to its enzymatic activity, SOD1 has been implicated in cellular signaling, RNA binding, ribosome biogenesis, and transcriptional regulation, with studies demonstrating its translocation to the nucleus in response to oxidative stress, where it modulates the expression of genes involved in stress resistance and genomic stability [21, 24].

These functions are tightly regulated by SOD1 structural integrity, metal occupancy, post-translational modifications (PTMs), and intracellular localization, making this antioxidant enzyme highly responsive to changes in the cellular environment [24]. Furthermore, under pathological conditions, the disruption of these regulatory mechanisms can promote structural destabilization, aberrant conformations, protein misfolding, and aggregation [6, 7, 13, 29]. Consequently, SOD1 shifts from a cytoprotective enzyme to a neurotoxic protein capable of impairing proteostasis, mitochondrial function, RNA metabolism, and inflammatory responses, ultimately contributing to neuronal dysfunction.

**Fig. 1:**
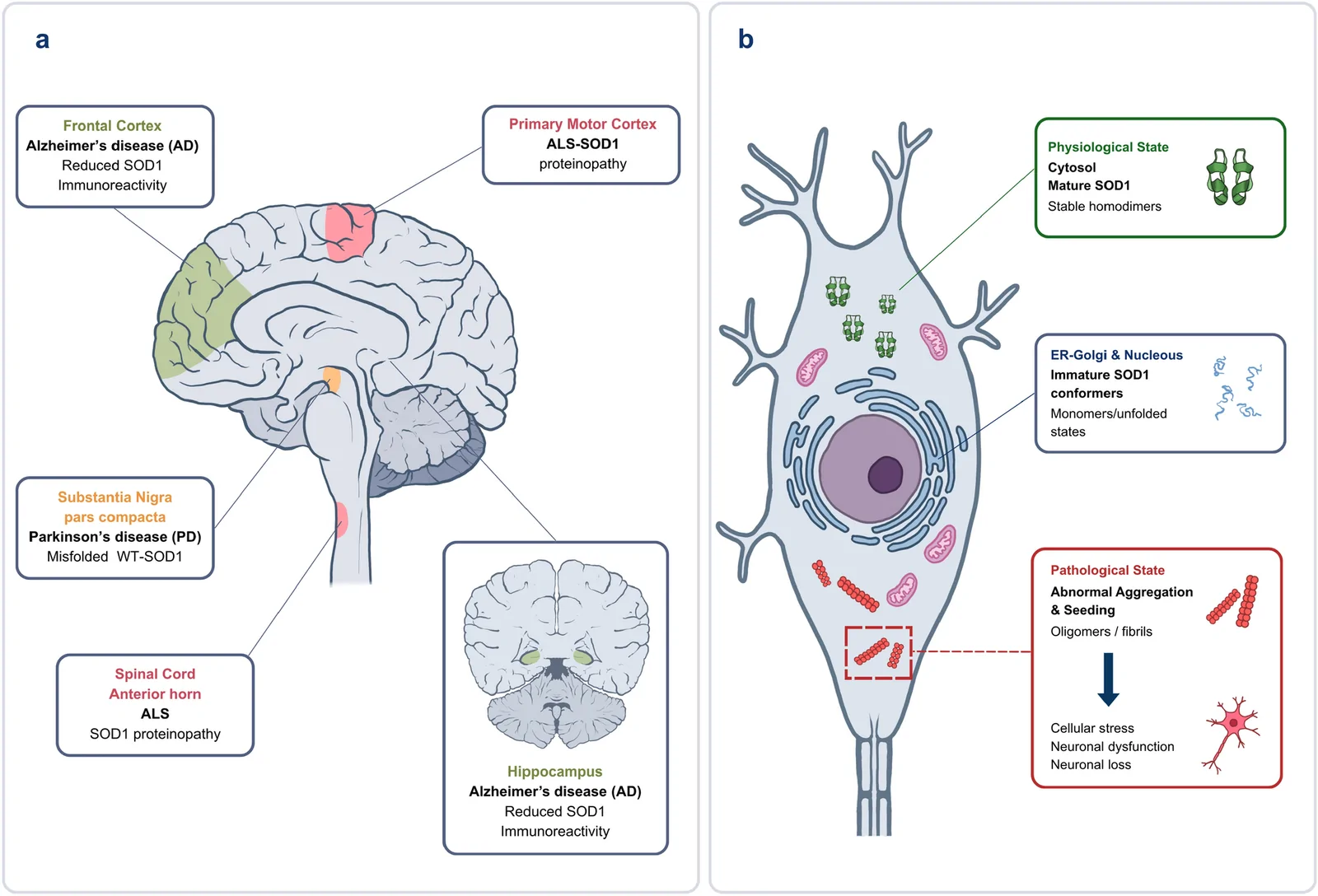
Regional distribution of physiological SOD1 and selective emergence of SOD1 alterations across the human central nervous system.** a** Physiological SOD1 is broadly expressed throughout the CNS under normal conditions. Disease-associated SOD1 alterations emerge selectively in anatomically vulnerable regions and differ according to the underlying neurodegenerative disorder. In amyotrophic lateral sclerosis (ALS), SOD1 proteinopathy predominantly affects the primary motor cortex and anterior horn motor neurons of the spinal cord. In Parkinson’s disease (PD), misfolded wild-type SOD1 has been reported in the substantia nigra pars compacta. In Alzheimer’s disease (AD), reduced SOD1 immunoreactivity has been observed in the frontal cortex, temporal cortex, and hippocampus, whereas the cerebellum remains relatively preserved. **b** Under physiological conditions, mature SOD1 is predominantly localized in the cytosol, whereas immature SOD1 conformers are associated with the endoplasmic reticulum-Golgi pathway and nuclear compartment during protein maturation. Under pathological conditions, conformational instability promotes SOD1 misfolding, ultimately contributing to neuronal dysfunction and loss. Illustration created by Leonardo Fogaça based on concepts developed by the authors

Although these mechanisms were first characterized in ALS following the identification of SOD1 mutations as a genetic cause of the disease [58], increasing evidence indicates that disturbances in SOD1 biology (i.e., altered expression, aberrant PTMs, conformational instability, and protein misfolding) are not restricted to ALS. They have also been reported in other neurodegenerative disorders, including PD and AD, supporting the concept that SOD1 may participate in convergent pathogenic pathways across neurodegeneration (Fig. 1A), while alterations in its intracellular localization and conformational state further contribute to its functional diversity (Fig. 1B) [16, 69].

Despite growing evidence that SOD1 pathology may extend beyond classical SOD1-associated motor neuron disease, its neuropathological significance in the human brain remains poorly defined. In particular, it is still unclear how robustly SOD1 misfolding or aggregation can be demonstrated in human brain tissue, whether its occurrence is associated with specific patterns of co-pathology involving other disease-associated proteins, and what these associations reveal about the mechanisms underlying pathological convergence across neurodegenerative disorders. The translational relevance of these findings also remains insufficiently explored, particularly regarding the potential of SOD1-centered pathological interactions to inform biomarker development and therapeutic strategies. In this review, we synthesize the available human neuropathological evidence for SOD1 misfolding and aggregation in the brain and examine its reported co-occurrence with TDP-43, tau, aSyn, amyloid-β, and other disease-associated proteins. We further evaluate the mechanistic interpretations emerging from these observations and consider their implications for biomarker development and therapeutic targeting, with the aim of defining what is currently established, what remains unresolved, and how SOD1-centered protein interactions may contribute to pathological convergence and heterogeneity across neurodegenerative diseases.

## Methodology

This review was conducted under the PRISMA Extension for Scoping Reviews guidelines [67], and a protocol outlining the objectives, eligibility criteria, and analytical strategy was developed a priori and registered on the Open Science Framework (OSF; https://osf.io/wykjm/overview?view_only=273016f100264c48a18893e4e2db46ea). Briefly, a systematic literature search was conducted in PubMed/MEDLINE and Web of Science to identify relevant studies published between December 2020 and December 2025. Studies were considered eligible if they investigated SOD1 misfolding, aggregation, pathological conformations, or PTMs in the context of neurodegenerative diseases. Experimental, clinical, and observational studies published in peer-reviewed journals were included, while reviews, editorials, opinion articles, and isolated clinical case reports were excluded. To fulfill the objective of this scoping review, we attempted to answer four main research questions: i) “*What neuropathological evidence supports SOD1 misfolding or aggregation in the brain?*”; ii) “*Which protein copathologies are reported to co-occur with SOD1 in brain tissue*?”; iii) “*What mechanistic insights have been proposed based on human evidence?”*; iv) “*What biomarker and therapeutic implications emerge from these findings?*”*.*

**Fig. 2:**
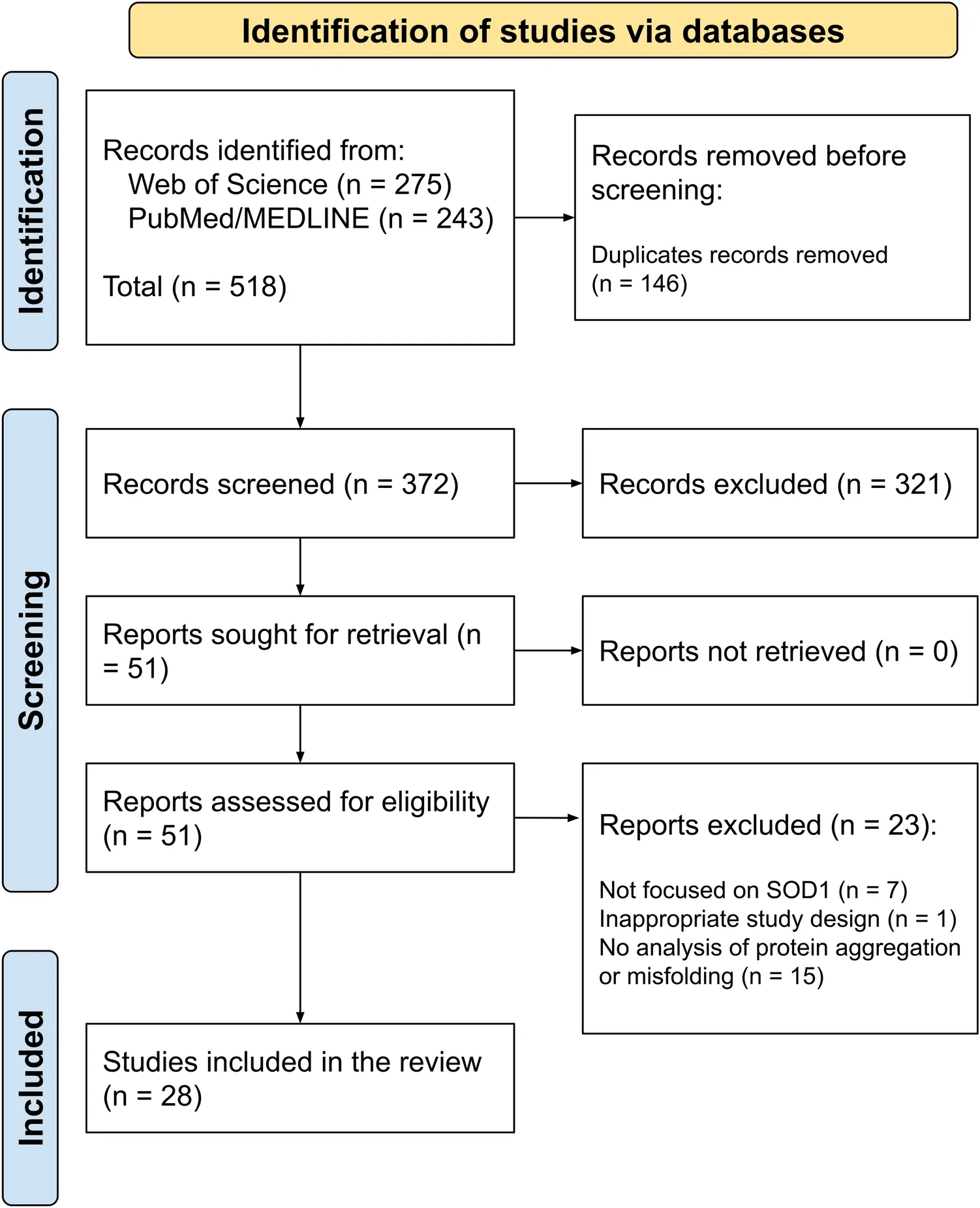
Flow diagram for study selection according to Systematic Reviews and Meta-Analysis (PRISMA) guidelines. Figure created by the authors

A total of 275 records were identified in the Web of Science Database and 243 in the PubMed/Medline, yielding 518 unique entries. After removing 146 duplicates, 372 records were screened by title and abstract by two independent reviewers. Full texts of 51 potentially eligible studies were retrieved and assessed in detail. At this stage, the articles were excluded if they did not focus on SOD1, if they presented inadequate study design, or if they lacked analyses of protein aggregation or misfolding. In total, 28 studies met all inclusion criteria and were incorporated into the final review (Fig. 2).

In this review, we organized the findings by disease type, brain region, SOD1 modification type, and reported copathologies. The experimental models were analyzed separately to distinguish mechanistic insights from direct evidence from human neuropathology. The narrative synthesis was complemented by summary tables that map the distribution of evidence and identify recurring mechanistic patterns and translational implications (Table 1).

**Table 1:** Characteristics of the studies included in this scoping review

Disease type	Study type	Main SOD1-related finding	Reported copathologies	References
AD	In vitro cellular models and human brain samples	Tau regulates SOD1 activity via lysosomal mTORC1 activity	Amyloid-β, tau	[46]
AD	Human cerebrospinal fluid	SOD1 was identified as part of a CSF proteomic signature associated with AD, clustering with tau and other neurodegeneration-related proteins, and supporting its potential as a biomarker of shared pathogenic mechanisms beyond ALS	Tau; PARK7 (DJ-1); YKL-40	[9]
ALS	Bioinformatic analysis	ALS mutations destabilize the SOD1 homodimer, alter metal-binding properties and favor formation of toxic trimers	None reported	[54]
ALS	Bioinformatic analysis	Systems biology analyses identified SOD1 as a central node linking oxidative stress, mitochondrial dysfunction, inflammation and apoptosis to molecular networks shared with other neurodegenerative diseases, highlighting predicted interactions with APP, APOE and SNCA	aSyn, APP, APOE	[65]
ALS	In vitro studies	N-terminal acetylation accelerates SOD1 amyloid formation without markedly destabilizing the apo protein	None reported	[34]
ALS	Bioinformatic analysis and in vitro cell models	Itraconazole promotes proteasome-mediated degradation of aggregation-prone mutant SOD1, reducing ubiquitin-positive inclusions and enhancing cellular protein quality control	None reported	[23]
ALS	Bioinformatic analysis and in vivo mice model	Epigenetic dysregulation in ALS corticospinal motor neurons altered SOD1-associated pathways involved in oxidative stress, proteostasis and axonal homeostasis, revealing coordinated regulation of SOD1, TDP-43 and FUS as potential drivers of selective neuronal vulnerability	TDP-43; FUS	[49]
ALS	Bioinformatic analysis	SOD1 occupies central molecular modules related to oxidative stress and proteostasis, linking ALS with other neurodegenerative diseases	TDP-43; FUS; tau; aSyn	[35]
ALS	In vitro cell models	Fisetin activates Nrf2-mediated autophagy, promoting degradation of mutant SOD1 aggregates and restoring proteostasis	p62/autophagy pathway	[71]
ALS	In vitro cell models	SOD1 is secreted through both free and extracellular vesicle-associated unconventional pathways, and ALS-linked mutations differentially modulate these secretion mechanisms according to their structural properties	None reported	[20]
ALS	In vitro cell models	Ibudilast activated TFEB-dependent lysosomal biogenesis and autophagy, promoting the simultaneous clearance of SOD1 and TDP-43 aggregates and supporting lysosomal modulation as a shared therapeutic strategy for ALS proteinopathies	TDP-43	[15]
ALS	In vitro cell models	Mutant SOD1 disrupted RNA metabolism and activated type I interferon signaling, revealing molecular cross-talk with TDP-43, FUS and hnRNPs that links proteostasis failure to neuroinflammation and neuronal vulnerability	TDP-43; FUS; hnRNPs	[32]
ALS	In vitro cell models	Misfolded SOD1 exposes normally buried residues that promote selective binding to TRAF6, influencing ubiquitination and aggregation	TRAF6	[27]
ALS	In vitro cell models	Stable soluble SOD1 trimers were identified as distinct toxic oligomeric species, and pharmacological or structural destabilization of trimer formation reduced cytotoxicity, highlighting SOD1 conformational states as therapeutic targets	None reported	[74]
ALS	In vitro cell and yeast models	Glycation promotes SOD1 oligomerization and aggregation, reduces enzymatic activity, enhances stress granule localization and induces TDP-43 pathology	TDP-43	[43]
ALS	Human CSF samples with a subset with post-mortem neuropathology	Approximately 14% of ALS patients exhibited aSyn seeding activity in CSF, supporting coexistence of aSyn pathology in a subset of ALS cases	aSyn	[62]
ALS	Human post-mortem motor cortex and spinal cord	Misfolded SOD1 from familial and sporadic ALS exhibits robust seeding activity, supporting prion-like propagation	None reported	[41]
ALS	Human post-mortem spinal cord	Disordered SOD1, phosphorylated TDP-43 and p62 coexist in ALS motor neurons, with SOD1 mutation status determining their patterns of co-deposition, colocalization and nucleocytoplasmic redistribution	TDP-43, p62	[68]
ALS	Human post-mortem motor cortex tissues	Quantitative neuropathological analysis revealed genotype-specific proteinopathy profiles in ALS, with SOD1-mutant cases showing predominant SOD1 inclusions and minimal TDP-43 pathology, supporting molecular stratification based on distinct proteopathic signature	TDP-43; p62; FUS	[45]
ALS/FTD	In vitro cell models	Mitoxantrone reduces both TDP-43 and SOD1 accumulation, partially restoring proteostasis and mitochondrial function	TDP-43; SQSTM1	[4]
ALS/FTD	In vitro cell models and in vivo mouse model	Selective HDAC6 inhibition with EKZ-438 markedly reduced misfolded SOD1, restored proteostasis and axonal transport, improved motor function, and simultaneously promoted clearance of TDP-43 aggregates, supporting a shared therapeutic strategy for ALS proteinopathies	TDP-43	[33]
ALS/FTD	In vitro cell models; in Vivo Zebrafish Model and human post-mortem spinal cord and brain	Specific tryptophan residues mediate TDP-43-induced SOD1 misfolding. Blocking these interactions reduces aggregation and toxicity	TDP-43	[53]
ALS and other proteinopathies	In vitro cell models	Pan-chaperone inhibition selectively promotes autophagic clearance of mutant SOD1 G93A while simultaneously targeting other aggregation-prone neurodegenerative proteins, highlighting shared proteostasis mechanisms across proteinopathies	APP; Tau; TDP-43 (shared proteostasis targets)	[19]
Hypoxic–ischemic encephalopathy (HIE)	In vitro, in vivo and post-mortem tissue	Acute hypoxic–ischemic injury triggered early SOD1 misfolding together with aSyn and prion protein aggregation across multiple CNS cell types, suggesting that acute brain injury can initiate proteopathic mechanisms shared with chronic neurodegenerative diseases	aSyn; PrP	[39]
Multiple neurodegenerative diseases	In vitro cell models and Postmortem brain and spinal cord tissues	PJA1 promoted ubiquitin-mediated proteasomal degradation of mutant SOD1 while simultaneously suppressing aggregation of TDP-43, tau and aSyn, identifying a shared proteostasis mechanism across multiple neurodegenerative proteinopathies	aSyn; TDP-43; tau	[72]
PD	Human substantia nigra tissue and in vivo mouse model	Wild-type SOD1 undergoes misfolding, altered metallation and abnormal PTMs in PD. Disordered SOD1 pathology induces dopaminergic neurodegeneration	aSyn (phosphorylated pS129)	[1]
Protein aggregation	In vitro studies	Lysine acetylation of SOD1 amyloid fibrils enhances their prion-like propagation properties	None reported	[11]
Synucleinopathies	Bioinformatic analysis	Network analyses positioned SOD1 as an indirect component of aSyn associated molecular networks, linking neuroinflammation, cellular stress and shared neurodegenerative pathways through predicted interactions with APP and inflammatory mediators	APP, aSyn	[64]

### SOD1 in the broader landscape of neurodegenerative proteinopathies

Growing evidence indicates that factors leading to structural destabilization represent a central event in neurodegenerative diseases in general, and also in SOD1-mediated pathogenesis. Although relatively infrequent, missense mutations are an obvious molecular factor affecting the structural stability of proteins. In this context, the analysis of a large dataset of ALS-associated missense variants suggests that mutation-induced protein destabilization is a major determinant of the pathogenicity of ALS-associated SOD1 missense variants, particularly within functionally important regions such as Cu^2^⁺-binding sites, leading to impaired SOD1 catalytic activity [54]. Consistently, 87.1% of the analyzed ALS-associated SOD1 variants are predicted to disrupt SOD1 homodimer stability, shifting the equilibrium toward the formation of toxic trimers [54].

Supporting the idea that destabilization precedes aggregation, recent studies have identified trimeric SOD1 species as critical intermediates linking conformational instability to both intracellular aggregation and intercellular propagation. The introduction of the trimer-destabilizing mutations N53I or D101I markedly reduces aggregation and oligomerization induced by the ALS-associated variants SOD1^G85R^ and SOD1^G93A^ [74]. In contrast, expression of the trimer-stabilizing mutant SOD1^G147P^ promotes aggregate formation. Furthermore, the stabilization of trimeric species enhances SOD1 uptake, whereas trimer-destabilizing mutations reduce both protein internalization and oligomerization [74]. Together, these findings support the view that SOD1 trimers are not merely transient intermediates in the aggregation pathway but may represent intrinsically pathogenic species that contribute to disease progression.

Additionally, structural studies have provided mechanistic insight into how destabilization may promote pathogenic interactions. Molecular dynamics simulations have shown that alterations in metal-dependent structural elements can modify the interaction properties of mutant SOD1. A recent study reported that the unfolding of loops IV and VII, which normally surround copper- and zinc-binding sites, changes the mode of interaction between SOD1 and the MATH domain of TRAF6 [27]. While well-folded SOD1 interacts predominantly through residues located at the native homodimer interface, disordered loop IV became a major contributor to TRAF6 binding following structural destabilization [27]. These findings suggest that mutation-induced conformational changes not only promote self-association but may also rewire protein interaction networks involved in ALS pathogenesis.

Notably, the genetic mutations are not the sole drivers of SOD1 destabilization. PTMs can also shift SOD1 toward aggregation-prone conformations. In this context, we recently demonstrated that glycation of recombinant human SOD1 by methylglyoxal (MGO) significantly increases the formation of SDS-stable oligomers, particularly trimers, while concomitantly reducing enzymatic activity [43]. Similarly, N-terminal acetylation accelerates the amyloid assembly of SOD1^wt^ without affecting its secondary structure or thermal stability [34]. The modification shortens the nucleation phase and increases fibril elongation rates by more than tenfold, while preserving the ability of acetylated and non-acetylated fibrils to cross-seed each other without significantly affecting fibril morphology.

Besides promoting aggregation, the structural alterations also influence the extracellular behavior of SOD1 by modulating its cellular trafficking. Distinct ALS-associated SOD1 variants differentially regulate unconventional protein secretion, with the D76Y variant reducing extracellular release, whereas the G93A and A4V variants enhance secretion [20]. These findings indicate that mutation-specific conformations determine the efficiency of pathogenic SOD1 export from cells. Given the growing evidence supporting the cell-to-cell transmission of misfolded proteins in neurodegenerative disorders [50], altered SOD1 secretion may represent an important mechanism linking intracellular misfolding to the propagation of pathology.

Collectively, these studies support a mechanism in which molecular factors, including genetics and PTMs, converge on a common pathogenic cascade involving structural destabilization, trimer formation, aberrant protein interactions, aggregation, and extracellular spread. This framework may help explain how structurally distinct SOD1 species ultimately contribute to a shared neurodegenerative phenotype. Furthermore, bioinformatic and systems biology approaches position SOD1 within molecular networks extending beyond ALS and intersecting with other neurodegenerative proteinopathies. Network-based analyses identify SOD1 as an indirectly connected node within aSyn-associated signaling pathways, biological functions, and protein interaction networks, suggesting a previously underrated relationship between these proteins at the molecular network level [64]. Additional analyses further reveal molecular connections linking SOD1 to proteins associated with AD, including amyloid precursor protein (APP) and apolipoprotein E (APOE) [65]. While APP is associated with SOD1-related signaling pathways, APOE emerged within interaction and co-expression networks, highlighting potential intersections between SOD1 biology, amyloid-related processes, and lipid metabolism.

Expanding these network-based observations, protein–protein interaction (PPI) analyses place SOD1 within interconnected molecular networks involved in oxidative stress, protein folding, proteostasis, lipid metabolism, apoptosis, and RNA processing [35]. Notably, SOD1 displayed direct or indirect associations with several genes and/or proteins implicated in distinct neurodegenerative disorders, including APP, APOE, SNCA, MAPT, TARDBP, and FUS. Collectively, these studies suggest that SOD1 participates in a broader molecular landscape of neurodegeneration, supporting the concept that pathogenic pathways traditionally associated with different proteinopathies may be more interconnected than previously thought.

### The relationship between SOD1 and TDP-43 pathology

Among the proteins linked to SOD1 in network analyses, TDP-43 has attracted particular attention due to its central role in ALS pathology. TDP-43 is a highly conserved, predominantly nuclear RNA-binding protein that regulates alternative splicing and other aspects of RNA metabolism through binding to UG-rich RNA sequences [8, 48, 66]. Under physiological conditions, TDP-43 forms dynamic nuclear homo-oligomers that are required for its splicing activity while simultaneously preventing aberrant self-association and pathological aggregation [2].

The experimental evidence indicates that SOD1 and TDP-43 are functionally connected under conditions of cellular stress. For example, methylglyoxal-induced metabolic stress promotes SOD1/TDP-43 interaction, supporting the concept that stress-dependent changes in SOD1 biology may facilitate crosstalk between these proteins [56]. Although multiple experimental studies support functional interactions between SOD1 and TDP-43, the neuropathological relevance and extent of their coexistence in human disease remain incompletely understood.

The neuropathological studies generally support the view that mutant SOD1-mediated ALS follows a pathogenic route distinct from the canonical TDP-43 proteinopathy observed in most ALS cases. Human post-mortem tissue analyses reveal mutation-dependent patterns of SOD1/TDP-43 copathology [68]. A 6.5-fold reduction in spinal motor neurons retaining normal nuclear TDP-43 localization is accompanied by the accumulation of phosphorylated TDP-43 (pTDP-43), a pathological marker of TDP-43 aggregation, which displays a diffuse cytoplasmic distribution in affected spinal motor neurons. In contrast, pTDP-43 inclusions are largely absent in SOD1-fALS despite the high burden of disordered mutant SOD1 pathology, whereas they are frequently observed in non-SOD1-fALS and sporadic ALS. Although disordered SOD1 pathology is detected across all ALS subtypes, co-occurrence analyses reveal limited overlap between mutant SOD1 and pTDP-43 pathology in SOD1-fALS motor neurons.

In contrast, wild-type disordered SOD1 and pTDP-43 frequently coexist in non-SOD1-fALS and sporadic ALS [68]. These findings suggest that mutant SOD1 aggregation drives neurodegeneration through mechanisms largely independent of TDP-43 pathology. Consequently, this may reflect convergent pathological processes affecting the same vulnerable neuronal populations, although the available neuropathological evidence does not establish whether these abnormalities are mechanistically linked or arise independently. This interpretation is further supported by the absence of detectable pTDP-43 pathology in ALS cases carrying SOD1 mutations, while pTDP-43 aggregation remains a prominent neuropathological feature of sporadic ALS and other ALS-TDP subtypes [45]. Together, these studies support the concept that mutant SOD1 and TDP-43 define distinct pathological signatures within the ALS spectrum.

Nevertheless, the apparent lack of neuropathological overlaps does not exclude the possibility of direct molecular interactions between these proteins. Indeed, beyond promoting SOD1 misfolding, experimental studies suggest that mutant SOD1 can influence TDP-43 phosphorylation and cytoplasmic redistribution, supporting the existence of functional crosstalk between these proteins before the development of overt protein inclusions [30, 36]. TDP-43 promotes the misfolding of endogenous WT SOD1 and induces aggregation of a SOD1^G85R^ reporter protein in human cell models, while the co-expression of both proteins synergistically promotes axonopathy in vivo. These effects depend on specific tryptophan residues, namely Trp68 and Trp172 in TDP-43 and Trp32 in SOD1, which mediate SOD1 misfolding, aggregation, and cross-seeding. These findings suggest that although SOD1 and TDP-43 copathology may not consistently co-localize in patient tissues, direct interactions between the two proteins can occur under specific conditions and may contribute to disease progression.

Additional evidence for convergence between SOD1- and TDP-43-associated pathogenic pathways has emerged from epigenetic and proteomic studies. The reduced levels of 5-methylcytosine were observed in corticospinal motor neurons from both hSOD1^G93A^ and prp-TDP-43^A315T^ mouse models, indicating a shared reduction in DNA methylation [49]. However, the two models exhibited opposite patterns of hydroxymethylation, with increased 5-hydroxymethylcytosine in hSOD1^G93A^ mice and reduced levels in TDP^43A315T^ mice, suggesting that distinct epigenetic programs may underlie these proteinopathies despite some shared molecular alterations.

Similarly, proteomic analyses have revealed substantial overlap between SOD1- and TDP-43-associated interaction networks. In a mouse model expressing human SOD1L^126delTT^ 392 mutant SOD1-interacting proteins are identified, the majority of which are associated with mRNA processing [32]. Among these interactors, 11% correspond to previously reported TDP-43-binding proteins and enrichment analyses link the shared interactome to ALS-related and other neurodegenerative pathways [32]. Together, these findings support a model in which SOD1 and TDP-43 may engage partially overlapping molecular networks despite exhibiting distinct neuropathological signatures.

### Connection between SOD1 and AD-related pathways

Accumulating evidence suggests that SOD1 also participates in molecular pathways associated with AD. Among these, functional interactions between SOD1 and tau provide a potential mechanistic link between redox regulation and AD-related neurodegeneration. Phosphorylation of SOD1 at Thr40 by the mTORC1 complex reduces its enzymatic activity, thereby modulating hydrogen peroxide production and influencing mitochondrial DNA synthesis [46]. Tau acts upstream by regulating lysosomal mTORC1 activity, which in turn controls SOD1 phosphorylation and inhibition [46].

In tau-deficient neurons, SOD1 activity remains elevated and less responsive to nutrient stimulation, whereas re-expression of tau restores mTORC1’s capacity to suppress SOD1 [46]. Thereby, maintaining redox equilibrium and mitochondrial replication. Furthermore, pathological conditions associated with AD, such as exposure to Aβ oligomers, decrease AKT and mTORC1 signaling activity in HEK293 cells and mouse neuron cultures, leading to a tau-dependent increase in SOD1 activity. Consistent with these experimental findings, elevated SOD1 activity was also detected in brain tissue from AD patients.

Clinical studies further support an association between SOD1 and AD-related pathology. Increased SOD1 abundance is detected in the cerebrospinal fluid (CSF) of patients with AD [9]. Proteomic analyses performed in three independent AD cohorts further demonstrated a positive correlation between CSF SOD1 levels and total tau (t-tau) concentrations. Together, these findings suggest that SOD1 dysregulation may extend beyond ALS and participate in molecular pathways linked to tau biology, mitochondrial homeostasis, and AD progression.

### Context-dependent associations between SOD1 and aSyn pathology

Similar connections have also been proposed between SOD1 and aSyn-associated disorders, particularly PD. A recent experimental model showed that impaired copper homeostasis promotes SOD1^wt^ misfolding and nigrostriatal degeneration in the absence of SOD1 mutations or aSyn pathology, suggesting that SOD1 dysfunction may contribute to Parkinsonian phenotypes independently of aSyn aggregation [59]. However, the relationship between SOD1 and aSyn-associated pathology appears to be highly context-dependent. The presence of aSyn pathology in ALS seems to be heterogeneous across clinical subtypes. Notably, Guamanian ALS-Parkinsonism–dementia complex (ALS-PDC), a historically endemic neurodegenerative syndrome affecting populations in Guam and other Western Pacific regions, exhibits neuropathological features that overlap with both ALS and Parkinsonism [31, 52]. In a cohort of 127 ALS patients (comprising sporadic, ALS-PDC, and familial forms), seed amplification assays detected seed-competent, fibrillar aSyn species in 14% of cerebrospinal fluid samples, while none of the control subjects without neurodegenerative disease tested positive [62]. Positivity rates were significantly higher in sporadic ALS compared with controls and were particularly elevated among ALS-PDC cases. In contrast, familial ALS associated with C9orf72 or SOD1 mutations exhibited markedly lower aSyn seeding activity.

While aSyn pathology appears to have limited involvement in SOD1-ALS, emerging evidence suggests that SOD1 dysregulation may contribute to the pathology of PD. Alterations in SOD1 copper-binding capacity, isoelectric point, and PTM profile have been identified in the substantia nigra pars compacta (SNpc) of PD patients [1]. Notably, postmortem SNpc tissue from individuals without SOD1 mutations exhibited widespread alterations in SOD1 PTMs, including marked reductions in O- and N-glycosylation and N-acetylglucosamination across four serine and asparagine residues. In addition, eight atypical PTMs previously associated with SOD1 structural disorder and aggregation in vitro were significantly enriched in PD tissue compared with controls. These findings were partially recapitulated in the SOD1 overproduction and copper knockdown (SOCK) mouse model, which combines reduced copper availability via Ctr1 knockdown with SOD1^wt^ overexpression. SOCK mice exhibited an altered SOD1 PTM profile, increased SOD1 inclusions within dopaminergic neurons and astrocytes of the SNpc, and motor impairment despite the absence of aSyn pathology.

While most studies have focused on the involvement of SOD1 in classical neurodegenerative disorders, emerging evidence suggests that SOD1 proteopathy may also occur in other neurological conditions characterized by severe cellular stress. In neonatal human hypoxic–ischemic encephalopathy (HIE), immunohistochemical analyses identified the concurrent presence of aSyn and SOD1 pathological species in affected brain tissue [39]. HIE cases exhibited increased numbers of cells positive for aggregated and nitrated aSyn in the cerebral cortex, substantia nigra, striatum, entorhinal cortex, and hippocampus when compared with non-HIE controls. Examination of the same tissues also revealed widespread oxidized/misfolded SOD1 immunoreactivity in neuronal and glial populations, including cortical neurons, astrocytes, oligodendrocytes, striatal neurons, hippocampal CA1 neurons, and cerebellar Purkinje cells. Distinct intracellular accumulations of oxidized/misfolded SOD1, including nuclear, cytoplasmic, and inclusion-like structures, were observed across several of these regions.

**Fig. 3:**
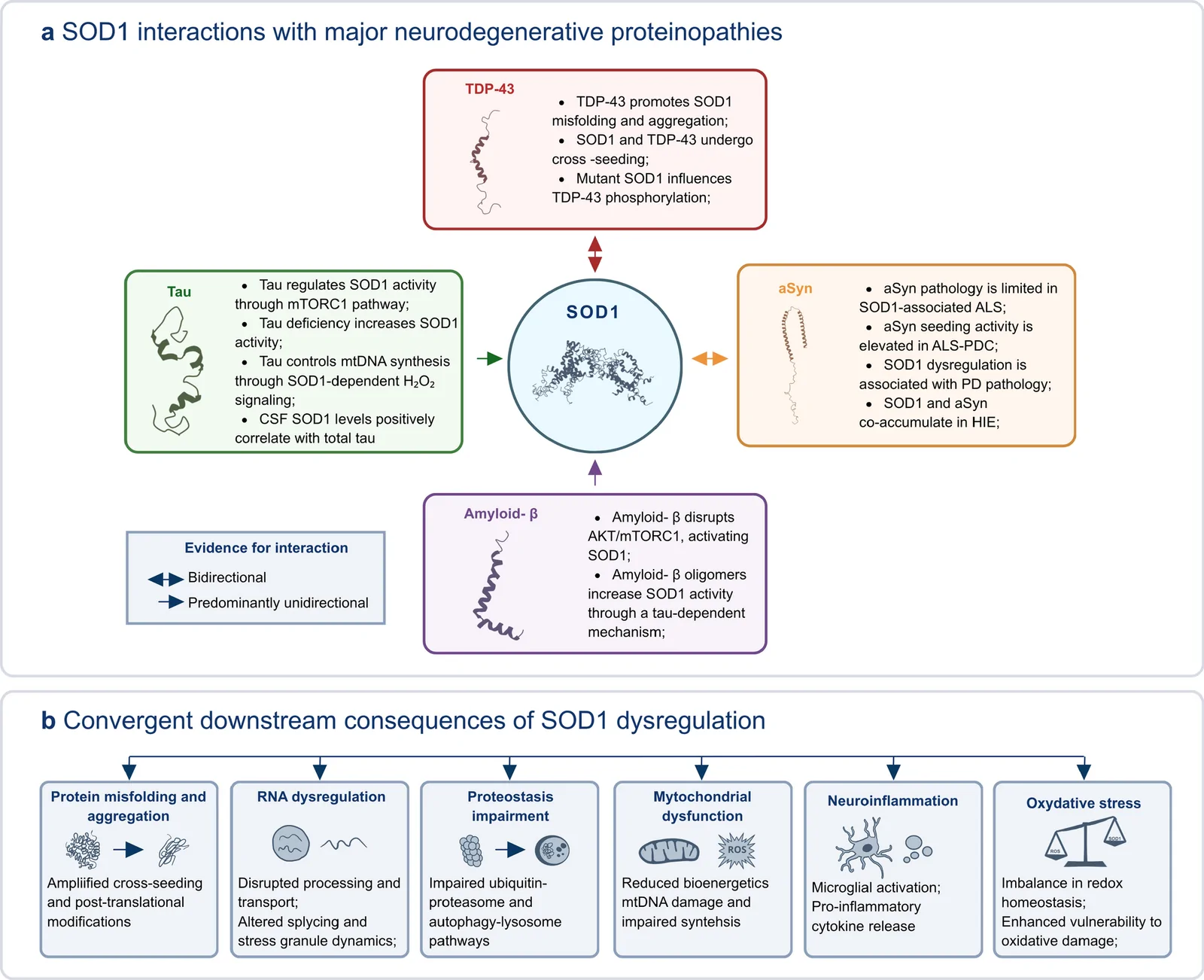
Molecular interactions between SOD1 and proteins implicated in neurodegenerative disorders. **a** Summary of the functional and pathological interactions between SOD1 and TDP-43, tau, amyloid-β, and alpha-synuclein (aSyn). TDP-43 promotes SOD1 misfolding and aggregation, while reciprocal effects of mutant SOD1 on TDP-43 phosphorylation support bidirectional crosstalk. Tau regulates SOD1 activity through mTORC1 signaling, whereas amyloid-β oligomers activate SOD1 through a tau-dependent mechanism involving AKT/mTORC1 disruption. The relationship between SOD1 and aSyn is context-dependent: aSyn pathology is limited in SOD1-associated Amyotrophic Lateral Sclerosis (ALS) but is more prominent in Amyotrophic Lateral Sclerosis-Parkinsonism-Dementia Complex (ALS-PDC), whereas SOD1 dysregulation is increasingly recognized in Parkinson’s disease (PD) and both pathological proteins co-occur in hypoxic-ischemic encephalopathy (HIE) [1, 30, 36, 39, 46, 53, 62, 64]. Arrows indicate the predominant direction of evidence reported in the literature, with double-headed arrows representing bidirectional interactions and single-headed arrows indicating predominantly unidirectional regulation. **b** Proposed downstream cellular consequences of SOD1 dysregulation, encompassing protein misfolding and aggregation, RNA dysregulation, proteostasis impairment, mitochondrial dysfunction, neuroinflammation, and oxidative stress, which converge to promote neuronal degeneration [29, 32, 35, 65]. Representative protein structures adapted from Protein Data Bank (PDB) entries: SOD1 (2NAM), tau (2MZ7), aSyn (1XQ8), TDP-43 (2N3X), and Aβ (1IYT). Illustration created by Leonardo Fogaça based on concepts developed by the authors

Collectively, these findings suggest that the relationship between SOD1 and aSyn pathology is highly context-dependent. Although previous studies have proposed direct interactions between these proteins, recent evidence indicates that their pathological overlap may vary substantially across neurodegenerative disorders. In particular, aSyn pathology appears to be limited in SOD1-associated ALS, whereas SOD1 dysregulation is increasingly recognized as a feature of PD. The main molecular interactions between SOD1 and the other aforementioned disease-associated proteins are summarized in Fig. 3.

### SOD1 as a biomarker

Given its involvement across multiple pathogenic pathways and its presence in diverse neurodegenerative disorders, considerable efforts have focused on evaluating SOD1 as a biomarker of disease-associated processes, including diagnosis, molecular classification, and monitoring of neurodegenerative pathology. Evidence discussed throughout this review suggests that SOD1 may be explored at multiple levels of biological complexity, including protein abundance, conformational state, post-translational modification profiles, and seeding activity.

Clinical studies have already identified alterations in SOD1 levels in biological fluids. For example, increased SOD1 abundance has been reported in the cerebrospinal fluid (CSF) of patients with AD, where SOD1 concentrations positively correlated with total tau levels [9]. Beyond protein abundance, neuropathological analyses have revealed distinct patterns of disordered SOD1 accumulation across ALS subtypes [68], suggesting that conformationally altered SOD1 species may provide disease-relevant information not captured by total protein measurements alone. In addition, disease-specific alterations in SOD1 PTMs have been identified in PD [1], supporting the possibility that SOD1 PTM signatures may serve as biomarkers of distinct neurodegenerative processes.

Among emerging approaches, seed amplification technologies have shown particular promise for detecting pathogenic protein conformers. Real-time quaking-induced conversion (RT-QuIC) assays detect SOD1 seeds in motor cortex and spinal cord samples at dilutions as high as 10⁻^5^, with seeding activity observed in both familial and sporadic ALS cases [41]. Notably, seeding activity was detected in both familial and sporadic ALS cases. These findings provide strong evidence that pathological SOD1 conformers represent disease-associated molecular species and support the use of seeding activity as a marker of underlying proteopathic processes. However, the current reliance on post-mortem neural tissues represents a major limitation for clinical translation, highlighting the need for studies investigating whether SOD1 seeds can be detected in more accessible biological fluids or peripheral samples.

Collectively, these observations highlight the growing potential of SOD1-based biomarkers and suggest that future approaches integrating measurements of protein abundance, structural state, post-translational modification patterns, and seeding activity may improve disease characterization, patient stratification, and monitoring of neurodegenerative processes. Such multidimensional strategies may ultimately provide greater sensitivity and disease specificity than conventional protein quantification alone. However, further studies are needed to establish their clinical utility, sensitivity, and specificity across different neurodegenerative disorders.

Lastly, beyond its diagnostic potential, SOD1 may also serve as a pharmacodynamic biomarker for therapeutic target engagement. Treatment with the brain-penetrant HDAC6 inhibitor EKZ-438 markedly reduced misfolded SOD1 accumulation in preclinical ALS models, accompanied by improvements in axonal transport, motor performance, and plasma biomarkers, including neurofilament light chain (NfL), nicotinamide phosphoribosyltransferase (NAMPT), and hepatocyte growth factor (HGF)[33]. These findings suggest that monitoring disease-associated SOD1 species may provide a valuable readout of therapeutic efficacy in addition to reflecting underlying proteostatic dysfunction.

## Therapeutic strategies targeting SOD1 pathology

### Targeting SOD1-TDP43 cross-seeding

The identification of a tryptophan-dependent cross-seeding mechanism between TDP-43 and SOD1 has revealed new opportunities for mechanism-based therapeutic intervention. Single-chain intrabodies targeting the RRM1 domain of TDP-43, which contains Trp172, reduce SOD1 misfolding by approximately two-fold in HEK293 cells [53]. In addition, treatment with 5-fluorouridine (5-FUrd), supplemented with uridine to mitigate toxicity, blocks the exposed Trp32 residue of SOD1 and significantly reduces TDP-43-induced SOD1 aggregation, supporting the concept that therapeutic strategies targeting Trp32 may attenuate SOD1-mediated neurotoxicity in ALS [53]. These findings indicate that disrupting specific molecular determinants involved in SOD1/TDP-43 cross-seeding may represent a promising strategy to limit the propagation of protein misfolding and downstream neurotoxicity in ALS.

Additional evidence supporting therapeutic modulation of the SOD1-TDP-43 axis comes from human induced pluripotent stem cell (hiPSC) models carrying TDP-43 mutations. Treatment with mitoxantrone, a synthetic nucleic acid intercalating agent and topoisomerase II inhibitor, reduces SOD1 expression to levels comparable to those observed in non-mutant cells, further supporting molecular crosstalk between TDP-43 pathology and SOD1 dysregulation [4].

### Enhancing autophagic and lysosomal clearance

Given the disruption of proteostasis observed in SOD1-related pathologies, modulation of the autophagic pathway has emerged as a potential therapeutic strategy. Autophagy degrades long-lived proteins, misfolded protein aggregates, and damaged organelles, thereby contributing to the maintenance of cellular proteostasis [38]. In this context, treatment with fisetin, a bioactive flavonoid known to regulate autophagic activity, in SOD1^G93A^-expressing cells shows a reduction in SOD1 aggregation in a dose-dependent manner and increases the expression of autophagy-related markers, including LC3-II, through activation of the Nrf2 pathway [71].

Similarly, treatment with AR12, an antiviral chaperone ATPase inhibitor, in combination with the antineoplastic agent neratinib, induces autophagosome formation, enhances autophagic flux, and reduces SOD1^G93A^ expression, further supporting the therapeutic potential of autophagy-inducing compounds to facilitate the clearance of pathogenic SOD1 species [19].

In addition to directly stimulating autophagy, therapeutic approaches targeting lysosomal biogenesis have also shown promise. Ibudilast, a non-selective phosphodiesterase (PDE) inhibitor with anti-inflammatory activity, suppresses mTORC1 signaling and promotes TFEB-dependent lysosomal biogenesis. In transfected HEK cells, ibudilast reduced the accumulation of both truncated TDP-43 and SOD1^G93A^ [15]. Collectively, these findings indicate that enhancing autophagic and lysosomal degradation pathways may represent an effective strategy to reduce the burden of aggregation-prone proteins and restore proteostasis in SOD1-associated neurodegeneration.

### Modulating proteostasis and protein quality control

In addition to autophagic and lysosomal pathways, several therapeutic approaches have focused on enhancing other components of the cellular protein quality control network. These mechanisms include molecular chaperone systems, axonal transport pathways, protein ubiquitination, and the ubiquitin–proteasome system (UPS), all of which contribute to maintaining proteostasis and clearing aggregation-prone proteins [37, 51].

Histone deacetylase 6 (HDAC6) is a predominantly cytoplasmic enzyme best known for its alpha-tubulin deacetylase activity and its role in regulating both proteostasis and intracellular transport. Treatment with EKZ-438, a next-generation HDAC6 inhibitor, reduces the accumulation of misfolded SOD1 and increases neuronal survival in primary motor neurons derived from SOD1^G93A^ rats [33]. In addition, EKZ-438 improves motor performance in SOD1G93A mice and enhances retrograde axonal transport, supporting HDAC6 inhibition as a promising strategy to mitigate SOD1-mediated neurodegeneration.

In addition to autophagy, the UPS represents another major intracellular pathway involved in protein quality control and has therefore emerged as a potential therapeutic target. The UPS is responsible for the selective degradation of misfolded, damaged, and other proteins involved in cellular pathways, thereby contributing to the maintenance of cellular proteostasis and homeostasis. Treatment with itraconazole enhances proteasomal activity, promoting the degradation of misfolded proteins and reducing the aggregation of SOD1^G37R^, aSyn^S87A^, and huntingtin in cellular models [23].

Similarly, Praja 1 (PJA1), a RING-finger E3 ubiquitin ligase, reduces the formation of SOD1^G93A^ aggregates and decreases the phosphorylation of both aSyn^wt^ and aSyn^A53T^ by promoting their ubiquitination [72]. Consistent with its role as an E3 ubiquitin ligase, PJA1 also undergoes self-ubiquitination and rapid proteasomal degradation. Analyses of human postmortem tissue further showed that intracytoplasmic hyaline inclusions in SOD1-ALS motor neurons, brainstem-type and cortical Lewy bodies in PD, and intranuclear inclusions in HD were not immunoreactive for PJA1. Collectively, these studies highlight the therapeutic potential of restoring protein quality-control mechanisms through complementary approaches that target HDAC6 signaling, proteasomal degradation, and protein ubiquitination. Such strategies may facilitate the clearance of pathogenic protein species and counteract the proteostatic imbalance characteristic of SOD1-associated disorders.

### Direct targeting of SOD1

The clinical validation of SOD1 as a therapeutic target has further reinforced interest in strategies aimed at modulating its pathological effects. Tofersen, an antisense oligonucleotide that promotes RNase H-mediated degradation of SOD1 mRNA and reduces SOD1 protein synthesis, is currently approved for treatment of adults with SOD1-associated ALS [12]. Long-term data from the phase 3 VALOR trial and its open-label extension provide further evidence that sustained SOD1 suppression can modify the course of SOD1-ALS. In the study, Tofersen treatment was associated with sustained reductions in cerebrospinal fluid SOD1 and NfL, consistent with prolonged target engagement and reduced neuroaxonal injury. Notably, in the faster-progressing subgroup, earlier treatment was associated with an approximately 3.4-year extension in event-free survival compared with delayed initiation [42].

Additionally, in a multicenter study of 18 patients receiving tofersen, a progressive reduction in CSF neurofilament levels during treatment was accompanied by increases in the neuroinflammatory markers SerpinA1 and CHI3L1 [61]. The authors proposed that the increase in inflammatory markers may reflect an immune response associated with prolonged antisense oligonucleotide exposure. These observations suggest that reducing SOD1 synthesis can attenuate neuroaxonal injury while leaving, or potentially reshaping, other disease-associated processes [61]. This distinction is particularly relevant in the context of SOD1 proteopathy, as lowering the production of the disease-driving protein may not directly eliminate pre-existing misfolded or aggregated species, alter their intercellular propagation, or reverse downstream changes in proteostasis, neuroinflammation, and other convergent proteinopathies. Thus, the clinical experience with tofersen not only establishes SOD1 as a therapeutically actionable target but also provides a rationale for complementary approaches directed at pathological SOD1 species and downstream mechanisms, including protein misfolding, aggregation, clearance, and propagation.

Given the central role of protein aggregation and seeded propagation in SOD1-mediated neurodegeneration, several therapeutic approaches have been developed to directly interfere with the formation, stability, and transmission of pathogenic SOD1 assemblies. PRG-A-04 was identified as a small molecule capable of reducing SOD1 aggregation by directly interacting with mutant SOD1. Biochemical analyses demonstrated a stronger binding affinity of PRG-A-04 for mutant SOD1 than for WT SOD1 [74]. In cultured cells, PRG-A-04 reduced SOD1 oligomerization induced by mutant SOD1^G85R^, SOD1^G93A^, and trimer-stabilizing SOD1 variants and prevented the co-accumulation of mutant and wild-type SOD1 within intracellular inclusions. Treatment with PRG-A-04 also reduced neuronal uptake of mutant SOD1 proteins and decreased subsequent oligomerization. In SOD1^G93A^ transgenic mice, PRG-A-04 reduced SOD1 inclusion bodies, insoluble SOD1 species, and SOD1 oligomerization, while increasing neuronal survival in the spinal cord. Improvements in motor performance were observed in rotarod and open-field assessments, and prolonged administration extended survival by approximately 25–30 days. Similar effects were obtained following oral administration of a bioavailable formulation of PRG-A-04 [74].

In addition to preventing aggregate formation, strategies aimed at reducing the propagation competence of existing SOD1 assemblies have also shown promise. Amyloid-targeting compounds that selectively acetylate lysine residues within fibrillar apo-SOD1, while sparing the native protein, increase the net negative charge of SOD1 aggregates and significantly reduce their seeding activity in vitro [11]. Moreover, acetylated fibrils exhibit diminished prion-like propagation in organotypic spinal cord cultures expressing mutant SOD1, resulting in a lower burden of induced inclusions. These findings suggest that modulation of the electrostatic properties of pathogenic SOD1 assemblies may represent a promising strategy to limit aggregate propagation and disease progression. These studies demonstrate that direct targeting of SOD1 aggregates can be achieved at multiple stages of the pathogenic cascade, including oligomer formation, aggregate stability, cellular uptake, and prion-like propagation.

Overall, the therapeutic approaches discussed in this review reflect the increasing shift toward mechanism-based strategies targeting distinct stages of SOD1-associated pathology. Rather than focusing on a single disease mechanism, current interventions aim to modulate multiple components of the pathogenic cascade, including SOD1/TDP-43 cross-seeding, impaired proteostasis, defective protein clearance, aggregate formation, and the propagation of misfolded species. These strategies include enhancing autophagic and lysosomal degradation, restoring UPS function, modulating protein quality control pathways, and directly targeting pathogenic SOD1 assemblies. Table 2 summarizes recent therapeutic approaches according to the molecular mechanisms they target, highlighting the diversity of strategies being explored to reduce SOD1 proteotoxicity and counteract neurodegenerative progression.

**Table 2:** Mechanism-based therapeutic approaches targeting the SOD1 pathogenic cascade

Pathogenic stage of SOD1 proteopathy	Therapeutic strategy	References
Aberrant SOD1 expression/production	Mitoxantrone-mediated normalization of SOD1 expression Tofersen-mediated suppression of SOD1 expression	[4] [42, 61]
SOD1 oligomerization and aggregate formation	Direct binding of mutant SOD1 by PRG-A-04	[74]
SOD1 seeding activity and prion-like propagation	Selective acetylation of fibrillar apo-SOD1 to reduce aggregate seeding competence Intrabodies targeting the TDP-43 RRM1 domain and Trp32-blocking compounds (5-FUrd)	[11, 53]
Impaired autophagic clearance	Fisetin-mediated activation of Nrf2 and autophagy AR12 combined with neratinib to enhance autophagic flux	[19, 71]
Lysosomal dysfunction and reduced protein clearance	Ibudilast-mediated suppression of mTORC1 and activation of TFEB-dependent lysosomal biogenesis	[15]
Defective proteostasis and axonal transport	HDAC6 inhibition using EKZ-438	[33]
Impaired ubiquitin–proteasome system (UPS) function and clearance of aggregation-prone proteins	Itraconazole-mediated enhancement of proteasomal activity Praja 1 (PJA1)-mediated ubiquitination of SOD1 and aSyn	[23, 72]

## Concluding remarks and outlook

The evidence summarized in this review highlights a growing paradigm shift in the understanding of SOD1 biology. Once regarded primarily as an antioxidant enzyme and later as a disease-specific protein implicated in familial ALS, SOD1 is increasingly recognized as a multifunctional protein that participates in broader proteopathic networks. Neuropathological studies demonstrate that pathological SOD1 species coexist with TDP-43, tau, aSyn, amyloid-β, and other aggregation-prone proteins across multiple neurodegenerative diseases, suggesting that SOD1 dysregulation may represent a convergent mechanism linking oxidative stress, proteostasis failure, and neuronal vulnerability. Nevertheless, whether these co-pathologies reflect direct molecular interactions, cross-seeding mechanisms, shared cellular stress responses, or independent parallel events remains largely unresolved.

Future studies should move beyond descriptive neuropathology to establish the mechanistic and temporal relationships between SOD1 and other pathogenic proteins. The integration of advanced human-based experimental models, spatial transcriptomics, spatial proteomics, single-cell multi-omics, high-resolution imaging, and biophysical approaches will be instrumental in determining how SOD1 contributes to disease initiation and progression. Likewise, standardized methodologies for detecting pathological SOD1 species in human tissues are essential for improving reproducibility and facilitating comparisons across studies. Ultimately, understanding SOD1 within the broader context of interacting proteinopathies may reshape current concepts of neurodegeneration, and elucidating these shared mechanisms will not only advance our understanding of disease convergence but may also uncover novel biomarkers and therapeutic strategies targeting common pathways underlying neurodegeneration.

## Data Availability

No datasets were generated or analyzed during the current study.
